# Mortality among hospitalized children with melioidosis in Thailand: a retrospective national database analysis (2015–2023)

**DOI:** 10.1016/j.lansea.2025.100707

**Published:** 2025-12-13

**Authors:** Phanthila Sitthikarnkha, Sirapoom Niamsanit, Leelawadee Techasatian, Suchaorn Saengnipanthkul, Kaewjai Thepsuthammarat, Pope Kosararaksa, Nattakarn Tantawarak, Punnathorn Auaboonkanok, Phenphitcha Pornprasitsakul, Rattapon Uppala

**Affiliations:** a Department of Pediatrics, Faculty of Medicine, Khon Kaen University, Khon Kaen, 40002, Thailand; b Clinical Epidemiology Unit, Faculty of Medicine, Khon Kaen University, Khon Kaen, 40002, Thailand

**Keywords:** Melioidosis, Hospitalization, Mortality, Children, Pneumonia

## Abstract

**Background:**

Pediatric melioidosis remains under-characterized nationally in Thailand, hindering triage and critical-care planning. We quantified epidemiology, complications, and mortality correlates among hospitalized children.

**Methods:**

Retrospective analysis of Thailand's National Health Security Office database from January 2015 to Dec 2023, including patients <18 years with principal melioidosis. Incidence and case-fatality rate were calculated. Mortality-related clinical characteristics were compared using descriptive statistics.

**Findings:**

Among 5044 admissions, 58.3% were male and 80.5% from the Northeast; annual incidence ranged 3.7–5.8 per 100,000, peaking in 2023. Median length of hospital stay was 11 days. Lower respiratory tract infection was the commonest localized focus (17.6%), followed by septic shock (2.9%). Organ dysfunction consisted of acute respiratory failure 3.2%, acute renal failure 2.3%, and disseminated intravascular coagulation (DIC) 1.7%. There were 2.3% required intubation with mechanical ventilation >96 h, and 2.2% needed renal replacement therapy. Overall, 84 children died (1.7%); deaths clustered in tertiary hospitals (71.4%). Mortality was markedly higher among children with septic shock, lower respiratory tract infection, and acute respiratory failure compared with children without these complications.

**Interpretation:**

National data identify a Northeast-weighted pediatric burden and a high-risk trajectory from pneumonia to acute respiratory failure, shock, and DIC. Embedding pediatric sepsis bundles with early melioidosis-active therapy and seasonal pediatric intensive care unit (PICU) surge/transfer protocols, may shorten time-to-treatment and reduce deaths substantially and equitably.

**Funding:**

This study was supported by the Fundamental Fund, 10.13039/501100004071Khon Kaen University, Thailand.

**Research in context**

**Evidence before this study**

We searched PubMed using combinations of the terms melioidosis OR *Burkholderia pseudomallei* AND (pediatric OR child OR children) AND (Thailand OR Thai) AND (mortality OR outcome OR epidemiology), and screened reference lists of key reviews; we included English and Thai studies reporting human data and prioritized peer-reviewed literature. Prior work establishes the global scale of melioidosis (≈165,000 cases and 89,000 deaths annually) and highlights endemicity across Southeast Asia, including Thailand. Existing evidence from Thailand documents substantial national burden with regional heterogeneity and under-recognition outside the Northeast, but pediatric-specific national analyses are scarce. Clinical and mechanistic reviews detail severe pulmonary disease, sepsis, and organ dysfunction as drivers of poor outcomes in melioidosis. Diagnostic studies show that culture, the reference standard has imperfect sensitivity; antigen-specific ELISAs (e.g., OPS-ELISA) and Thai-validated real-time PCR assays can provide earlier confirmation in critically ill patients. Therapeutic evidence from Thailand established ceftazidime as mortality-reducing intensive therapy for severe disease, while long-running resistance surveillance from Northeast Thailand has reported stable susceptibility to first-line agents with rare resistance. Pediatric series, principally from Australia and single-center Thai cohorts, demonstrate heterogeneous presentations across settings, but do not quantify national pediatric risk with contemporary Thai administrative data.

**Added value of this study**

Using Thailand's National Health Security Office database (2015–2023), we assembled the national cohort of hospitalized children with melioidosis to date (N = 5044), mapped incidence trends and geography (with a clear Northeast concentration), and Described clinical factors related with in-hospital death, most notably acute respiratory failure, septic shock, and disseminated intravascular coagulation (DIC), based on observed disparities between children who survived and died, signals that are clinically recognizable and immediately actionable in pediatric pathways. Our analysis aligns reporting with STROBE and bridges clinical epidemiology with pragmatic health-system levers (triage, rapid diagnostics, surge capacity), thereby addressing a documented gap in Thai pediatric melioidosis evidence.

**Implications of all the available evidence**

Our national data identify pediatric melioidosis as a seasonally intensified and geographically concentrated disease in Thailand, with the highest burden in the Northeast region and peak admissions during the rainy season. The findings show that mortality remains strongly associated with acute respiratory failure, septic shock, and DIC, underscoring the need for timely recognition and supportive management of these complications. Strengthening critical-care preparedness, and seasonal surge capacity in high-incidence provinces may help reduce preventable pediatric deaths from melioidosis in Thailand.

## Introduction

Melioidosis is an infectious disease caused by *B. pseudomallei*, which can result in significant morbidity and mortality, particularly in pediatric populations.^1^ Thailand is a long-standing hotspot where melioidosis is an important cause of death nationwide and shows marked regional heterogeneity, reflecting environmental suitability, exposure patterns, and laboratory capacity.^2^ Children affected by melioidosis may present with a range of clinical manifestations, including pneumonia and septicemia. The clinical manifestations of melioidosis are heterogeneous and can mimic other bacterial infections, which makes diagnosing melioidosis more challenging.^3^ An interdisciplinary approach involving physicians, microbiologists, and radiologists is essential for accurate diagnosis and timely initiation of appropriate therapy.^4^^,^^5^ The presentation of melioidosis with shock constitutes a life-threatening emergency requiring immediate and aggressive intervention, including proper antibiotics, adequate fluid resuscitation, and supportive care.^6^^,^^7^

Several factors have been identified as associated with mortality in pediatric patients hospitalized with melioidosis. Particularly, the presence of septic shock or severe sepsis complications at admission significantly correlates with increased risk of death. Additionally, clinical and laboratory findings such as abnormal liver function tests, elevated lactate levels, and leukocytosis serve as important associated factors.^3^ Children with underlying comorbidities, including malnutrition, immunosuppression, or chronic pulmonary conditions, may also face higher mortality risks.^8^ Delays in administering proper antibiotic therapy can also lead to poor outcomes.^6^ Early identification of high-risk individuals enables clinicians to optimize treatment strategies, thereby improving prognosis. Continuous monitoring of clinical signs and laboratory parameters, coupled with prompt antibiotic administration and effective management of sepsis-related complications, constitutes essential guidelines to reduce mortality rates among pediatric melioidosis patients.^7^

Understanding the incidence, clinical outcomes, and mortality factors of pediatric melioidosis in Thailand is essential for developing effective clinical guidelines. Although comprehensive, nationwide clinical data remain limited, our study utilized the National Health Security Office database to provide a national overview of pediatric melioidosis. The research aimed to evaluate epidemiological patterns, disease trends, and factors associated with mortality in hospitalized children diagnosed with melioidosis.

## Methods

This study was a retrospective observational analysis utilizing data from a comprehensive national database in Thailand. The study focused specifically on children under 18 years of age who were hospitalized with a diagnosis of melioidosis from January 2015 to December 2023. The post-audit dataset was obtained from the Thai National Health Security Office (NHSO), which maintains anonymized patient information under regulatory approval. The NHSO oversees reimbursement for healthcare facilities that provide services to Thai citizens under the Universal Coverage Scheme. Thailand's healthcare system comprises three primary schemes: the Universal Coverage Scheme for the uninsured, the Social Security Scheme for private sector employees, and the Civil Servant Medical Benefit Scheme for government employees and their dependents. Among these, the Universal Coverage Scheme is the predominant program, covering approximately 72% of the population.^9^ This study was reported following the Strengthening the Reporting of Observational Studies in Epidemiology (STROBE) checklist for observational studies.

### Study population

The data of hospitalized patients less than 18 years old under the universal health coverage scheme, including principal diagnoses, co-morbidities, complications, and procedures, were reviewed by NHSO upon discharge for reimbursement purposes. Diagnoses were classified using the International Statistical Classification of Diseases and Related Health Problems, 10th Revision, Thai Modification (ICD-10-TM). Procedures were documented using the International Statistical Classification of Diseases and Related Health Problems, 9th Revision, Clinical Modification (ICD-9-CM).

In Thailand, the diagnosis of melioidosis was confirmed using various methods, including bacterial culture, serological tests like the indirect hemagglutination assay (IHA) and ELISA (enzyme-linked immunosorbent assay), as well as polymerase chain reaction (PCR) results.^10^ This study included data on children under 18 years with principal diagnosis of melioidosis from ICD-10-TM codes: 1) A24.1: Acute and fulminant melioidosis refer to cases with clinical pneumonia or septicemia caused by *B. pseudomallei* infection; 2) A24.2: Subacute and chronic melioidosis involve infection with *B. pseudomallei* persisting for weeks to months and typically include abscess formation; 3) A24.3: Other melioidosis refers to cases infected by *B. pseudomallei* that are not classified as subacute or chronic; and 4) A24.4: Melioidosis, unspecified, when organ-specific details are provided elsewhere.^11^ Data that did not meet the ICD-10-TM diagnosis criteria were excluded from the study.

Demographic data, including age, gender, hospital region, hospital level, and date of admission, were obtained from the NHSO database. Co-morbidities refer to diagnoses identified prior to hospitalization. Each hospitalization record in the NHSO database represents a single admission episode. Because unique patient identifiers were not accessible, repeat admissions for the same child could not be linked and were analyzed as separate hospitalizations, consistent with standard national database methodology. Cases with co-infections or concurrent severe bacterial or viral infections were retained to reflect the real-world clinical spectrum of pediatric melioidosis. Only records that did not meet ICD-10-TM diagnostic criteria for melioidosis were excluded from analysis. The site of infection was extracted from the principal diagnosis and complications using ICD-10-TM, focusing on 1) septic shock (R57.2); 2) lower respiratory tract infections, including pneumonia (J17 and J18), lung abscess (J85), parapneumonic effusion (J90), and empyema thoracis (J86); 3) intraabdominal infections, including splenic abscess (D73.3) and liver abscess (K75); and 4) infections of the skin and subcutaneous tissue (L01-L08). Children with serologically positive results for melioidosis but without localized organ-specific bacterial culture were classified as having no localized organ infection.

The complications related to melioidosis involving organ dysfunction were classified by ICD-10-TM as follows: 1) acute respiratory failure (ICD-10-TM codes: J80 and J96.0); 2) acute renal failure (ICD-10-TM code: N17); 3) acute hepatic failure (ICD-10-TM code: K72.0); and 4) disseminated intravascular coagulation (DIC), which indicates hematologic involvement (ICD-10-TM code: D65). We extracted data on continuous mechanical ventilation lasting more than 96 consecutive hours (ICD-9-CM code 96.72) to assess the severity of respiratory failure, as well as the need for renal replacement therapy (ICD-9-CM codes 39.95 for hemodialysis and 54.98 for peritoneal dialysis) to determine the severity of acute renal failure.

The duration of a hospital stay is determined by calculating the number of days between the admission date and the discharge date, without regard to the patient's discharge status. To determine mortality outcomes, discharge status is classified into patients who deteriorate and die as the death group and those who recover as the alive group.

### Statistical analyses

This study used Stata version 18 (Stata Corp LLC, College Station, TX, US) for statistical analysis. Continuous variables, such as median length of stay, were presented as median with interquartile range (IQR), whereas categorical variables were shown as numbers and percentages. Mortality-related clinical characteristics were compared using Chi-square test. The incidence of melioidosis hospitalization was calculated and expressed as rates per 100,000 people in the population. The mortality rate was presented as per 1000 admissions. A p-value of less than 0.05 was considered statistically significant.

### Ethical statement

This study was reviewed and approved by the Khon Kaen University Human Research Ethics Committee, under reference number HE681327. The requirement for informed consent was waived because the study used unidentified personal data and was conducted retrospectively, in accordance with the principles of the Declaration of Helsinki.

### Role of funding source

The funders did not participate in designing the study, collecting or analyzing data, deciding to publish, or preparing the manuscript.

## Results

### Incidence rate of hospitalization

**Fig. 1:**
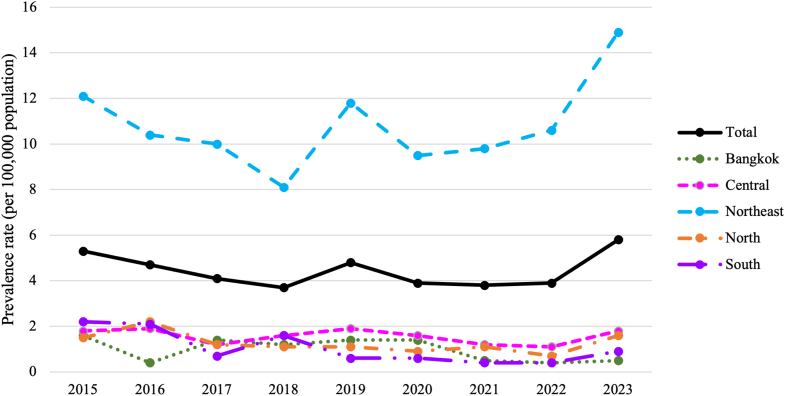
**The incidence rate of hospitalization among children with melioidosis infection during 2015–2023**.

**Fig. 2:**
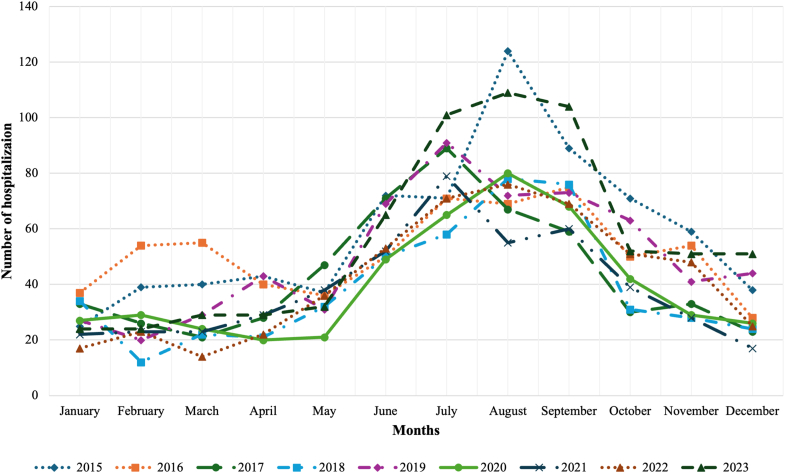
**Number of hospitalizations among children with melioidosis infection by month from 2015 to 2023**.

During the study period, there were 5044 hospitalized children diagnosed with melioidosis. Of these, 2941 children (58.3%) were male, and nearly half were between 5 and 12 years of age (2410 children; 47.8%). The annual incidence rates fluctuated between 3.7 per 100,000 persons in 2018 and 5.8 per 100,000 persons in 2023 (Fig. 1). Hospital admission numbers were highest each year during July to September, which corresponds to the peak rainfall period in Thailand (Fig. 2). The northeastern region exhibited the highest prevalence of melioidosis infections, accounting for 4060 children (80.5%). The secondary hospital level had the highest number of admissions, with 3391 children (67.2%). Most children were diagnosed with melioidosis unspecified accounting for 4252 cases (84.3%). Acute and fulminant melioidosis were the most common diagnoses among children who died at hospital discharge (54 children; 64.3%). This study found that 2361 children (46.8%) have at least one medical co-morbidity. There were 3548 children (70.3%) who did not exhibit any localized organ infection. Among those with culture-confirmed localized infections, lower respiratory tract infections were the most prevalent, affecting 885 children (17.6%). There were 144 children (2.9%) who developed septic shock from melioidosis infection (Table 1). The median length of stay was 11 days (IQR 6–15).

**Table 1:** Baseline characteristics of hospitalized children with melioidosis during 2015–2023, comparing survivors and non-survivors.

Characteristics	Total (N = 5044)		Alive (N = 4960)		Death (N = 84)		p-value
	n (%)	95% CI	n (%)	95% CI	n (%)	95% CI	
**Sex**							0.500
Male	2941 (58.3)	56.9, 59.7	2889 (58.3)	56.9, 59.6	52 (61.9)	50.7, 72.3	
Female	2103 (41.7)	40.3, 43.1	2071 (41.8)	40.4, 43.1	32 (38.1)	27.7, 49.3	
**Age group**							<0.001
<1 yr	69 (1.4)	1.1-1.7	62 (1.3)	1.0, 1.6	7 (8.3)	3.4, 16.4	
1–<5 y	1484 (29.4)	28.2, 20.7	1464 (29.5)	28.3, 30.8	20 (23.8)	15.2, 34.4	
5–<12 y	2410 (47.8)	46.4, 49.2	2380 (48.0)	46.6, 49.4	30 (35.7)	25.6, 46.9	
12–<18 y	1081 (21.4)	20.3, 22.6	1054 (21.3)	20.1, 22.4	27 (32.1)	22.4, 43.2	
**Region**							0.006
Bangkok	80 (1.6)	1.3, 1.9	80 (1.6)	1.3, 2.0	0 (0)	0	
Central	454 (9)	8.2, 9.8	449 (9.1)	8.3, 9.9	5 (6)	1.9, 13.4	
Northeast	4060 (80.5)	79.4, 81.6	3996 (80.6)	79.4, 81.7	64 (76.2)	65.7, 84.8	
North	235 (4.7)	4.1, 5.3	230 (4.6)	4.1, 5.3	5 (6.0)	1.9, 13.4	
South	215 (4.3)	3.7, 4.9	205 (4.1)	3.6, 4.7	10 (11.9)	5.9, 20.8	
**Hospital level**							<0.001
Primary	366 (7.3)	6.6, 8	365 (7.4)	6.7, 8.1	1 (1.2)	0.1, 6.5	
Secondary	3391 (67.2)	65.9, 68.5	3368 (67.9)	66.6, 69.2	23 (27.4)	18.2, 38.2	
Tertiary	1274 (25.3)	24.1, 26.5	1214 (24.5)	23.3, 25.7	60 (71.4)	60.5, 80.8	
Private	13 (0.3)	0.1, 0.4	13 (0.3)	0.1, 0.5	0 (0)	0	
**Diagnosis**							<0.001
Acute and fulminant melioidosis	650 (12.9)	11.9, 13.8	596 (12)	11.1, 12.9	54 (64.3)	53.1, 74.5	
Subacute and chronic melioidosis; Other melioidosis; and Melioidosis, unspecified	4394 (87.1)	86.2, 88.1	4364 (88)	87.1, 88.9	30 (35.7)	25.6, 46.9	
**Site of infection**							
Septic shock	144 (2.9)	2.4, 3.6	85 (1.7)	1.4, 2.1	59 (70.2)	59.3, 79.7	<0.001
Lower respiratory tract infection	885 (17.6)	16.5, 18.6	819 (16.5)	15.5, 17.6	66 (78.6)	68.3, 86.8	<0.001
**Organ involvement**							
Acute respiratory failure	161 (3.2)	2.7, 3.7	99 (2.0)	1.6, 2.4	62 (73.8)	63.1, 82.8	<0.001
Acute renal failure	117 (2.3)	1.9, 2.8	76 (1.5)	1.2, 1.9	41 (48.8)	37.7, 60	<0.001
Disseminated intravascular coagulation	86 (1.7)	1.4, 2.1	40 (0.8)	0.6, 1.1	46 (54.8)	43.5, 65.7	<0.001
**With co-morbidities**	2361 (46.8)	45.4, 48.2	2280 (46)	44.6, 47.4	81 (96.4)	89.9, 99.3	<0.001

### Complications related to melioidosis infection

A total of 252 children (5%) developed organ dysfunction, with acute respiratory failure being the most common, affecting 161 children (3.2). Of these, 114 children required endotracheal tube intubation with invasive mechanical ventilation for over 96 consecutive hours. Among the age groups, adolescents aged 12 to less than 18 years exhibited the highest prevalence of endotracheal intubation, accounting for 41 children (36%). Acute renal failure represented the second most common organ dysfunction, affecting 117 children (2.3%). Of these, 110 children (2.2%) required renal replacement therapy, with the highest prevalence among children aged 5 to less than 12 years (40 children, 36.4%) and 12–18 years (39 children, 35.5%) (Table 2).

**Table 2:** The number of intubations with mechanical ventilation more than 96 h and renal replacement therapy of hospitalized children diagnosed with melioidosis, divided by age group.

Age group	Need intubation with mechanical ventilation more than 96 h (N = 114)	Need Renal replacement therapy (N = 110)
<1 year	7 (6.1)	7 (6.4)
1–<5 years	30 (26.3)	24 (21.8)
5–<12 years	36 (31.6)	40 (36.4)
12–<18 years	41 (36)	39 (35.5)

Note. Data presented as number (percentage).

### Mortality rate

**Fig. 3:**
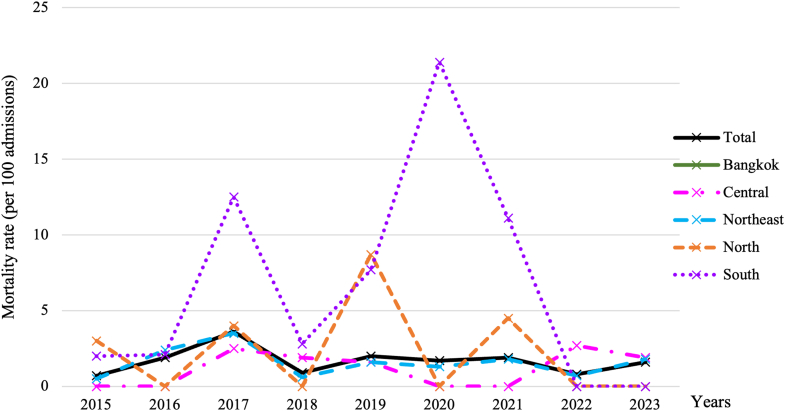
**Annual mortality rate per 100 admissions of children diagnosed with melioidosis infection**.

A total of 84 hospitalized children died from melioidosis infection. The annual case fatality rate varied, ranging from 0.7% in 2015 to 3.6% in 2017. The annual mortality rate per 100 admissions is illustrated in Fig. 3. The median length of stay for this group was 3.5 days (IQR 1–8). Children diagnosed with septic shock and lower respiratory tract infections demonstrated elevated mortality rates in comparison to those without these conditions. Acute respiratory failure emerged as the primary end-organ failure, correlating with heightened mortality risk relative to cases lacking such complications (Table 1).

## Discussion

This national analysis characterizes the spatial concentration and clinical determinants of pediatric melioidosis in Thailand from 2015 to 2023, showing that admissions were overwhelmingly drawn from the Northeast region and that mortality clustered in children with acute respiratory failure, alongside septic shock and DIC, aligning with the recognized lethality of severe pulmonary melioidosis and pediatric sepsis in endemic settings.^12^^,^^13^ Thai and regional pediatric series consistently flag pneumonia and bacteremia as the dominant pathways to poor outcomes, contextualizing our finding within a broader clinical pattern.^4^^,^^14^ Geographical concentration of cases in Northeast Thailand in our data mirrors national epidemiology and surveillance summaries, underscoring a true endemic gradient rather than a referral artifact.^2^^,^^10^ Biological plausibility for the high-risk respiratory phenotype is supported by virulence determinants of *B. pseudomallei*, including capsular polysaccharide and variable LPS, that amplify host inflammatory injury in lung tissue.^15^^,^^16^ These clinical and mechanistic considerations justify an aggressive, guideline-concordant sepsis bundle and early institution of melioidosis-active parenteral therapy for children presenting with fulminant respiratory involvement.^13^^,^^17^

Children presenting with septic shock in our dataset had markedly higher mortality compared with those without shock, in keeping with prior Thai and regional cohorts linking shock to mortality in melioidosis.^18^^,^^19^ We observed a strong mortality association with disseminated intravascular coagulation, which coheres with mechanistic studies showing intense coagulation activation and impaired anticoagulant pathways in melioidosis sepsis.^20^ Endothelial perturbation and hemostatic imbalance are further supported by studies demonstrating elevated von Willebrand factor with relative ADAMTS13 deficiency and platelet-related vulnerability in melioidosis.^21^^,^^22^ Our findings highlight that mortality signals such as septic shock, respiratory failure, and coagulopathy often occur in children with underlying comorbidities. Although specific antimicrobial and microbiological data were unavailable, these results reinforce that prompt recognition, timely initiation of melioidosis-active therapy, and appropriate management of comorbidities such as diabetes to improving pediatric outcomes in endemic regions. Together, these data prioritize early recognition of shock and coagulopathy and escalation to pediatric critical care for bundled resuscitation, hemodynamic support, and timely source control.^13^ Our findings suggest that any child with melioidosis presenting with bacteremia should be managed in a high-dependency or pediatric intensive care unit (PICU) setting due to the high risk of rapid deterioration.

Within our study, adolescents (12–<18 years) constituted the largest share of endotracheal intubations, aligning with observations that pediatric melioidosis is clinically heterogeneous across ages and may skew toward severe pulmonary disease in some settings.^4^^,^^23^ Australian pediatric data emphasize cutaneous disease predominance in otherwise healthy children, highlighting that our Thai profile, with substantial respiratory support requirements, reflects setting-specific exposure routes and pathogen–host interactions.^23^ Given this variability, triage algorithms in endemic hospitals should explicitly weight hypoxemia and work of breathing to trigger early PICU referral and definitive airway management.^13^

Lower respiratory tract infection was the leading culture-confirmed focus among children with organ-localized disease in our data, a pattern consistent with long-term clinical series in endemic regions.^24^ Pneumonia in melioidosis can progress rapidly with multilobar involvement and high mortality, demanding prompt empiric coverage and close monitoring for ARDS and ventilatory failure.^4^ Environmental triggers, especially intense rainfall, likely increase inhalational exposure and case severity, supporting seasonal preparedness for PICUs in endemic provinces.^25^

Acute kidney injury and the need for renal replacement therapy, while less frequent than respiratory failure in our cohort, nonetheless tracked with severe sepsis burden and resource-intensive care.^13^ Institutional adult-leaning Thai cohorts similarly link multiorgan dysfunction to mortality, reinforcing the value of early renal involvement and standardized criteria for initiating dialysis in pediatric melioidosis.^19^ Where available, integration of pediatric sepsis pathways with nephrology consult triggers may shorten time to organ support and improve hemodynamic stability.^13^

Our admission rates rose to 5.8 per 100,000 children in 2023 with a heavy Northeast concentration, echoing national analyses that identify Northeast Thailand as the epicenter of the country's melioidosis burden.^10^ Model-based global maps suggest that Thailand lies within a high-suitability belt where true incidence likely exceeds reported counts, supporting our interpretation that the pediatric caseload we observed underestimates community burden.^26^ Documented under-reporting in southern provinces further cautions that pediatric melioidosis is more widespread than routine surveillance implies, warranting strengthened pediatric case detection.^27^ The persistently higher incidence of pediatric melioidosis in the Northeast and, to a lesser extent, the North of Thailand likely reflects a combination of environmental, healthcare access, and diagnostic factors. The Northeast region's soil composition, rainfall patterns, and rice-farming exposure create ecological conditions that strongly favor *B. pseudomallei* persistence and transmission.^10^^,^^28^ Between 2015 and 2023, we observed a progressive increase in incidence, with the highest annual burden recorded in 2023. This upward trend is likely multifactorial and may reflect intensified surveillance activities, broader access to diagnostic testing, and environmental changes associated with rainfall variability.^10^ In support of a climatic influence, our data demonstrated that hospital admission numbers were highest each year during July to September, which corresponds to the peak rainfall period in Thailand. This consistent seasonal pattern suggests that precipitation-related factors may substantially modulate transmission dynamics and exacerbate disease burden during the rainy season. The transient mortality increase around 2020 is likely attributable to COVID-19-related disruptions in healthcare access, delayed presentation, and strained critical-care capacity. Conversely, the observed decline in mortality rates likely reflects improvements in early recognition, adherence to pediatric sepsis protocols, and wider access to melioidosis-active antimicrobials such as ceftazidime and carbapenems.^29^

The preponderance of admissions at secondary hospitals with a concentration of deaths at tertiary centers in our study likely reflects referral of the most unstable children, paralleling national pediatric septic shock patterns clustered in higher-level facilities.^30^ Planning for surge capacity and interfacility transfer protocols is essential in the rainy season to minimize delays to airway management, vasopressors, and definitive antimicrobials.^13^ Thai susceptibility data confirm the predictable resistance profile of *B. pseudomallei*, guiding empiric selection and stewardship once cultures return.^31^

Early ceftazidime was historically shown to reduce mortality in severe melioidosis and remains a cornerstone for initial parenteral therapy in resource-constrained endemic hospitals.^17^ Network meta-analysis indicates that carbapenems may confer additional survival benefits in high-risk presentations, which is relevant to ventilated children or those in shock.^32^ Embedding pediatric Surviving Sepsis Campaign bundles with empiric melioidosis-active regimens and early source control offers the best chance to blunt the fatal trajectory we observed.^13^

Our Northeast-weighted pediatric caseload reinforces the need for community-level risk-reduction measures (e.g., protective footwear in fields and avoidance of unboiled water) tailored to children's daily activities.^28^ Evidence from Thailand demonstrates ingestion and rainfall-linked exposures as independent risks, motivating household water safety messaging and rainy-season precautions for families.^28^ Confirmed contamination of drinking water with *B. pseudomallei* in Thai provinces strengthens the rationale for point-of-use boiling advisories during peak months.^33^ At the system level, national briefings should prioritize laboratory training to reduce misidentification of *B. pseudomallei*, upgrade PICU readiness in endemic regions, and ensure timely access to ceftazidime/carbapenems.^34^

A principal strength of this study is its national scope across Thailand's Universal Coverage database over nine years, with a large pediatric sample size analyzed and reported under STROBE guidance to enhance transparency and comparability.^35^ However, several limitations should be acknowledged. The analysis relied on ICD-10-TM coding for disease phenotypes and complications, which may lead to under-ascertainment of culture-negative cases and variable use of serological testing across facilities.^34^ This study used post-audit administrative data from the National Health Security Office, in which hospital claims are reviewed for accuracy before reimbursement. Diagnoses are based on clinical presentation and supporting laboratory evidence according to national case definitions; however, the database does not record the specific diagnostic method (culture, serology, or PCR) applied in each case, precluding stratification by diagnostic modality. Our national administrative dataset also lacked detailed clinical and microbiological information, including underlying diabetes mellitus, timing of symptom onset, specific organ abscess types, and standardized severity scores (e.g., qSOFA). Furthermore, antimicrobial therapy data—covering drug selection, duration, and susceptibility testing—were unavailable because these parameters are not captured in reimbursement claims. As a result, our analysis could not assess treatment adequacy, which is a recognized determinant of outcome in melioidosis. These limitations may constrain causal interpretation of mortality predictors; however, the dataset's national coverage and standardized coding still provide a robust overview of epidemiological patterns and high-risk clinical features. We also lacked standardized time-to-antibiotic and physiologic severity metrics at presentation, which may bias toward more severe cases referred to tertiary centers.^13^ Country-level surveillance studies indicate under-reporting outside traditional hotspots, suggesting that our pediatric burden estimates are conservative and calling for enhanced capture of non-Northeast provinces.^27^ Future prospective studies are warranted to incorporate detailed treatment data, antibiotic susceptibility profiles, and timing of therapy to more accurately elucidate drivers of mortality in pediatric melioidosis. Linking clinical outcomes with microbiological and pharmacologic data would enable clearer assessment of treatment adequacy and resistance emergence. In parallel, community-based and environmental studies could identify modifiable risk factors, such as water exposure, soil contact, and behavioral practices, that predispose children to infection in endemic areas. Together, these research avenues will complement our national database findings and inform more targeted prevention and early-intervention strategies.

Our national analysis identifies acute respiratory failure, septic shock, and DIC as dominant mortality signals in hospitalized Thai children with melioidosis, mapping closely to known pathophysiology and high-risk pulmonary presentations in endemic contexts. Translating these signals into practice entails rigorous pediatric sepsis bundles, early melioidosis-active antibiotics, and upgraded PICU pathways in endemic provinces during high-rainfall periods. Public-health actions focused on household water safety and field exposure mitigation can plausibly reduce pediatric incidence.

## Contributors

PS: Conceptualization, Methodology, Analysis, Supervision, Validation, Data Curation, Project administration, Writing-original draft, Writing review and Editing; RU: Conceptualization, Methodology, Analysis, Supervision, Validation, Data Curation, Project administration, Writing-original draft, Writing review and Editing; KT: Analysis, Validation, Data Curation, Writing-original draft, Writing review; SN: Methodology, Analysis, Visualization, Writing-original draft, Writing review and Editing; SS: Visualization, Writing-original draft, Writing review and Editing; LT: Visualization, Writing-original draft, Writing review and Editing; NT: Visualization, Writing-original draft, Writing review and Editing; PA: Visualization, Writing-original draft, Writing review and Editing; PK: Visualization, Writing-original draft, Writing review and Editing; PP: Visualization, Writing-original draft, Writing review and Editing. All authors reviewed the manuscript and approved the final version of the manuscript. All authors had full access to all the data in the study and accept responsibility for the decision to submit for publication.

## Data sharing statement

The datasets generated and/or analyzed during the current study are not publicly available but are available from the corresponding author (RU) upon request.

## Declaration of interests

The authors declare no competing interests.

## References

[1] Cheng A.C., Currie B.J. (2005). Melioidosis: epidemiology, pathophysiology, and management. Clin Microbiol Rev.

[2] Hantrakun V., Kongyu S., Klaytong P. (2019). Clinical epidemiology of 7126 melioidosis patients in Thailand and the implications for a national notifiable diseases surveillance system. Open Forum Infect Dis.

[3] Currie B.J., Dance D.A.B., Cheng A.C. (2008). The global distribution of Burkholderia pseudomallei and melioidosis: an update. Trans R Soc Trop Med Hyg.

[4] Meumann E.M., Cheng A.C., Ward L., Currie B.J. (2012). Clinical features and epidemiology of melioidosis pneumonia: results from a 21-year study and review of the literature. Clin Infect Dis.

[5] Wiersinga W.J., Virk H.S., Torres A.G. (2018). Melioidosis. Nat Rev Dis Primers.

[6] Cheng A.C., Fisher D.A., Anstey N.M., Stephens D.P., Jacups S.P., Currie B.J. (2004). Outcomes of patients with melioidosis treated with meropenem. Antimicrob Agents Chemother.

[7] Princess I., Ebenezer R., Ramakrishnan N., Daniel A.K., Nandini S., Thirunarayan M.A. (2017). Melioidosis: an emerging infection with fatal outcomes. Indian J Crit Care Med.

[8] Warapitiya D.S., Subasinghe S., de Silva R.F., Piyarisi D.L., Jayatilleke K. (2021). Severe sepsis with multiorgan failure due to melioidosis: a lesson to learn. Case Rep Med.

[9] Sumriddetchkajorn K., Shimazaki K., Ono T., Kusaba T., Sato K., Kobayashi N. (2019). Universal health coverage and primary care, Thailand. Bull World Health Organ.

[10] Hinjoy S., Hantrakun V., Kongyu S. (2018). Melioidosis in Thailand: present and future. Trop Med Infect Dis.

[11] (2016). ICD-10-TM. http://thcc.or.th/ebook1/2016/mobile/index.html#p=1.

[12] Gassiep I., Armstrong M., Norton R. (2020). Human melioidosis. Clin Microbiol Rev.

[13] Weiss S.L., Peters M.J., Alhazzani W. (2020). Surviving sepsis campaign international guidelines for the management of septic shock and sepsis-associated organ dysfunction in children. Intensive Care Med.

[14] Lumbiganon P., Chotechuangnirun N., Kosalaraksa P., Teeratakulpisarn J. (2011). Localized melioidosis in children in Thailand: treatment and long-term outcome. J Trop Pediatr.

[15] Burtnick M.N., Heiss C., Roberts R.A., Schweizer H.P., Azadi P., Brett P.J. (2012). Development of capsular polysaccharide-based glycoconjugates for immunization against melioidosis and glanders. Front Cell Infect Microbiol.

[16] Norris M.H., Schweizer H.P., Tuanyok A. (2017). Structural diversity of Burkholderia pseudomallei lipopolysaccharides affects innate immune signaling. PLoS Negl Trop Dis.

[17] White N.J., Dance D.A., Chaowagul W., Wattanagoon Y., Wuthiekanun V., Pitakwatchara N. (1989). Halving of mortality of severe melioidosis by ceftazidime. Lancet.

[18] Churuangsuk C., Chusri S., Hortiwakul T., Charernmak B., Silpapojakul K. (2016). Characteristics, clinical outcomes and factors influencing mortality of patients with melioidosis in southern Thailand: a 10-year retrospective study. Asian Pac J Tropical Med.

[19] Chayangsu S., Suankratay C., Tantraworasin A., Khorana J. (2024). The predictive factors associated with In-Hospital mortality of melioidosis: a cohort study. Medicina (Kaunas).

[20] Wiersinga W.J., Meijers J.C.M., Levi M. (2008). Activation of coagulation with concurrent impairment of anticoagulant mechanisms correlates with a poor outcome in severe melioidosis. J Thromb Haemostasis.

[21] Birnie E., Claushuis T.A.M., Koh G.C.K.W. (2019). Thrombocytopenia impairs host defense against burkholderia pseudomallei (Melioidosis). J Infect Dis.

[22] Birnie E., Koh G.C.K.W., Löwenberg E.C. (2017). Increased Von Willebrand factor, decreased ADAMTS13 and thrombocytopenia in melioidosis. PLoS Neglected Trop Dis.

[23] McLeod C., Morris P.S., Bauert P.A. (2015). Clinical presentation and medical management of melioidosis in children: a 24-Year prospective study in the Northern Territory of Australia and review of the literature. Clin Infect Dis.

[24] Stewart J.D., Smith S., Binotto E., McBride W.J., Currie B.J., Hanson J. (2017). The epidemiology and clinical features of melioidosis in Far North Queensland: implications for patient management. PLoS Neglected Trop Dis.

[25] Currie B.J., Jacups S.P. (2003). Intensity of rainfall and severity of melioidosis, Australia. Emerg Infect Dis.

[26] Limmathurotsakul D., Golding N., Dance D.A. (2016). Predicted global distribution of Burkholderia pseudomallei and burden of melioidosis. Nat Microbiol.

[27] Kaewrakmuk J., Chusri S., Hortiwakul T. (2023). Under-reporting cases and deaths from melioidosis: a retrospective finding in Songkhla and Phatthalung province of southern Thailand, 2014–2020. Trop Med Infect Dis.

[28] Limmathurotsakul D., Kanoksil M., Wuthiekanun V. (2013). Activities of daily living associated with acquisition of melioidosis in northeast Thailand: a matched case-control study. PLoS Neglected Trop Dis.

[29] Chandna A., Bonhoeffer M., Miliya T., Suy K., Sao S., Turner P. (2021).

[30] Niamsanit S., Sitthikarnkha P., Techasatian L., Saengnipanthkul S., Uppala R. (2024). Epidemiology and outcomes of septic shock in Thai children: a nationwide retrospective study from 2015 to 2022. Crit Care.

[31] Wuthiekanun V., Amornchai P., Saiprom N. (2011). Survey of antimicrobial resistance in clinical Burkholderia pseudomallei isolates over two decades in Northeast Thailand. Antimicrob Agents Chemother.

[32] Anothaisintawee T., Harncharoenkul K., Poramathikul K. (2023). Efficacy of drug treatment for severe melioidosis and eradication treatment of melioidosis: a systematic review and network meta-analysis. PLoS Negl Trop Dis.

[33] Limmathurotsakul D., Wongsuvan G., Aanensen D. (2014). Melioidosis caused by Burkholderia pseudomallei in drinking water, Thailand, 2012. Emerg Infect Dis.

[34] Lau S.K.P., Sridhar S., Ho C.-C. (2015). Laboratory diagnosis of melioidosis: past, present and future. Exp Biol Med (Maywood).

[35] Elm E.V., Altman D.G., Egger M. (2007). The strengthening the reporting of observational studies in epidemiology (STROBE) statement: guidelines for reporting observational studies. PLoS Med.

